# *De novo *identification of LTR retrotransposons in eukaryotic genomes

**DOI:** 10.1186/1471-2164-8-90

**Published:** 2007-04-03

**Authors:** Mina Rho, Jeong-Hyeon Choi, Sun Kim, Michael Lynch, Haixu Tang

**Affiliations:** 1 Department of Computer Science, Indiana University, Bloomington, IN 47405, USA; 2 Center for Genomics and Bioinformatics, Indiana University, Bloomington, IN 47405, USA; 3 School of Informatics, Indiana University, Bloomington, IN 47408, USA; 4 Department of Biology, Indiana University, Bloomington, IN 47405, USA

## Abstract

**Background:**

LTR retrotransposons are a class of mobile genetic elements containing two similar long terminal repeats (LTRs). Currently, LTR retrotransposons are annotated in eukaryotic genomes mainly through the conventional homology searching approach. Hence, it is limited to annotating known elements.

**Results:**

In this paper, we report a *de novo *computational method that can identify new LTR retrotransposons without relying on a library of known elements. Specifically, our method identifies intact LTR retrotransposons by using an approximate string matching technique and protein domain analysis. In addition, it identifies partially deleted or solo LTRs using profile Hidden Markov Models (pHMMs). As a result, this method can *de novo *identify all types of LTR retrotransposons. We tested this method on the two pairs of eukaryotic genomes, *C. elegans *vs. *C. briggsae *and *D. melanogaster *vs. *D. pseudoobscura*. LTR retrotransposons in *C. elegans *and *D. melanogaster *have been intensively studied using conventional annotation methods. Comparing with previous work, we identified new intact LTR retroelements and new putative families, which may imply that there may still be new retroelements that are left to be discovered even in well-studied organisms. To assess the sensitivity and accuracy of our method, we compared our results with a previously published method, LTR_STRUC, which predominantly identifies full-length LTR retrotransposons. In summary, both methods identified comparable number of intact LTR retroelements. But our method can identify nearly all known elements in *C. elegans*, while LTR_STRUCT missed about 1/3 of them. Our method also identified more known LTR retroelements than LTR_STRUCT in the *D. melanogaster *genome. We also identified some LTR retroelements in the other two genomes, *C. briggsae *and *D. pseudoobscura*, which have not been completely finished. In contrast, the conventional method failed to identify those elements. Finally, the phylogenetic and chromosomal distributions of the identified elements are discussed.

**Conclusion:**

We report a novel method for de novo identification of LTR retrotransposons in eukaryotic genomes with favorable performance over the existing methods.

## Background

Mobile genetic elements (MGEs, also called transposable elements, TEs), which can transpose from one location to another within the genome, are known to be one of the causes of large scale genome reorganization [1]. According to the mechanism of their transposition, MGEs are usually classified into two broad categories: *retroelements *(or class I elements), which are transposed through the reverse transcription of an RNA template (*retrotransposition*), and *DNA transposons *(or class II elements), which are transposed through a classical DNA "cut-and-paste" transposition model. MGEs have attracted the attention of evolutionary biologists in studying their interactions with the host species [2], especially in the post-genome era when more and more eukaryotic genomes are sequenced. The conventional approach to annotating MGEs in genomic sequences is based upon homology searching against a well-updated library of known MGEs, e.g. Repbase [3], using a fast searching program, e.g. RepeatMasker [4]. This approach, however, is limited to annotating those known MGE families, and thus cannot identify new elements. Furthermore, it sometimes even overlooks known elements, because the repetitive nature of MGE elements may confuse the statistical methods (e.g. E-values) that are commonly used in genome annotation [5].

In a pioneer paper, Bao and Eddy described a *de novo *approach to automated annotation of repeat elements in a genome [6]. Their program RECON clustered BLAST hits from self-comparison of a single genome and reported the repeat elements that appear many times in similar copies in the genome. Since then, several software tools have been developed with improved speed and performance over RECON, e.g. RepeatScout [7], PILER [8], and a combined method [9]. All these methods described above, however, attempted to identify repeat elements based on their copy numbers in a genome, thus facilitating identification of general repeat elements. Many MGEs indeed appear high copies in the host genome because of their transposition activity. But some MGE families have low copy numbers in some genomes. Furthermore, there exist other types of repeat elements than MGEs. For example, many low copy repeats (LCRs) in mammalian genomes are induced by segmental duplications [10]. Although these LCRs follow a completely different duplication mechanism from MGEs, there is often no clear distinction in copy numbers between these two classes of repeats. As a result, successful identification of new MGEs by these bioinformatics approaches requires subsequent manual inspection and experimental validation [11]. Recently, a new computational method was proposed that identified genome-specifically inserted sequences using multiple alignment of closely related genomes [12]. This new method does not rely on the copy number of the repeat elements to identify them, but does not attempt to distinguish different classes of repeats either.

In this paper, we adopt a different *de novo *approach to identifying mobile genetic elements, which is based on common structural models of specific MGE families, rather than their copy numbers in a genome. As an initial step of this approach, we concentrate on one class of mobile genetic elements, LTR-retrotransposons, which share a unique structural feature, two long terminal repeats (LTRs) that are longer than 100 bp and play a key role in their transposition. LTR retrotransposons and endogenous retroviruses have partially overlapping gene organizations, and thus are thought to have the same origin. Since two LTRs of a single LTR retrotransposon have identical sequences at the time of integration, dating the transposition event of a LTR retrotransposon can be achieved reliably by computing the sequence similarity of its two LTRs [13]. Therefore, LTR retrotransposons become an ideal subject for phylogenetic analysis. Computational screening of LTR retrotransposons has been done extensively in several eukaryotic genomes, e.g. *C. elegans *[14], *D. melanogaster *[15,16], mouse [17] and rice [18]. Software tools, such as LTR_STRUC [19], and a newly developed one [20] were developed to speed up the screening process. However, they were based on sequence characteristics derived from known LTR retroelements. Because of the high divergence of LTR retrotransposons [21], there are likely new elements still to be identified, even in these well-studied model organisms.

We propose here a *de novo *computational method for LTR retrotransposon identification that consists of three steps. In the first step, we identify only young and intact LTR retrotransposons, i.e. those elements associated with pairs of LTRs with high identity (e.g. > 80%). This problem can be formulated as finding two highly similar subsequences with a distance ranging typically from 1000 to 20000 bases in a given genomic sequence. We used an approximate string matching technique, based on the suffix array data structure, to solve this problem. In addition, the structure of retroelements is inspected by the occurrences of common protein domains. In the second step, we identify solo LTRs, i.e. the unpaired LTRs resulting from recombination between LTR retrotransposons, by first applying the BAG sequence clustering algorithm [22] to cluster LTRs identified in the previous step, and then searching against the whole genome using sequence profile Hidden Markov Models (pHMMs) built from these LTR sequence clusters. Finally, we identify old and intact LTR retrotransposons with LTR pairs of low identities (e.g. < 80%) by a phylogenetic analysis of identified LTR elements.

We implemented our method in a software package using C++ and perl, and tested it on two eukaryotic genomes, *C. elegans *and *D. melanogaster*. We chose these genomes for initial testing because they have been well studied so that we can compare our results with the previous known ones and those identified by LTR_STRUC [19]. It turns out that our *de novo *method identified almost all of the previously known elements, whereas LTR_STRUC missed about 1/3 of them, although both methods report comparable number of retroelements. This indicates our method has a higher sensitivity over the existing method. In addition to known elements, our method identified some new intact LTR retrotransposons and several putative new families of LTR retrotransposons. These are particularly encouraging results, for these two genomes have been well studied. In order to obtain a larger evolutionary picture of their transpositions, we also analyzed two additional genomes, *C. briggsae *and *D. pseudoobscura*, each closely related to one of the two model genomes. From the phylogenetic analysis of the identified elements, we find clear evidence that some LTR retrotransposon families are specific to single species within a genus, whereas some others are active across both genomes. We also analyzed the distribution of chromosomal locations of identified LTR retrotransposons. Consistent with previous reports, we observed that there were more LTR retrotransposons existing in heterochromatic regions than in euchromatic regions, implying that active mobile genetic elements might contribute significantly to the formation of heterochromatin.

## Results and discussion

### Identification of intact and solo LTR retroelements

Following the algorithms described in the Methods section, we implemented a program package for *de novo *LTR retrotransposon identification. We then applied our program to identify LTR retrotransposons in four genomes. Totals of 58, 33, 686, and 65 intact LTR retrotransposons were found in the *C. elegans*, *C. briggsae*, *D. melanogaster*, and *D. pseudoobscura *genomes, which were classified into 37, 19, 113, and 41 clusters, respectively (Table 1). We note that these clusters represent putative families of retroelements. Below we discuss the details of our findings in each of these four genomes.

#### Comparison with existing methods

**Figure 1:**
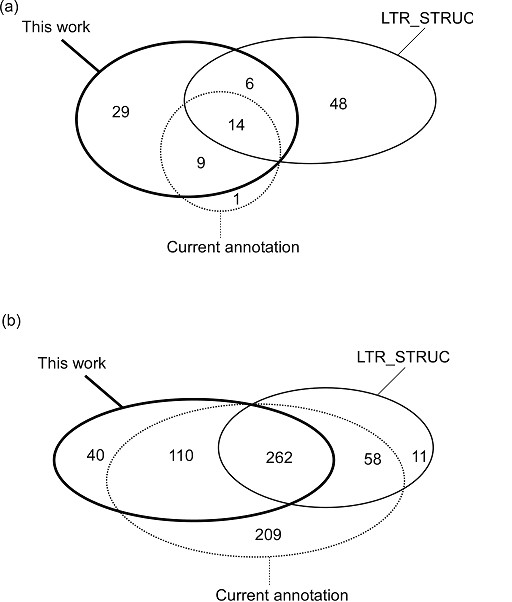
Comparison of identified LTR retrotransposons by our method and LTR_STRUC. (a) Venn diagram illustration of the comparison in *C. elegans*. Our method identified 23 out of 24 known elements, whereas LTR_STRUC identified 14. (b) Venn diagram illustration of the comparison in the *D. melanogaster*. Using only intact LTR retrotransposons, our method identified 418 out of 639 annotated elements, whereas LTR_STRUC identified 320. (c) Venn diagram illustration of the comparison in the *D. melanogaster*. Using both intact and solo LTR retrotransposons, our method identified 569 out of 639 annotated elements, whereas LTR_STRUC identified 320.

We first compared the results of our method with LTR_STRUC [19], a widely applied program for LTR retroelements. Figure 1(a) shows Venn diagram of the known intact LTR retroelements [14], and the intact retroelements identified by LTR_STRUC and our method in the *C. elegans *genome, respectively. The results show that both our method and LTR_STRUC report comparable number of retroelements (58 for our method and 68 for LTR_STRUC). Our method identified almost all (23/24) known intact LTR retroelements, whereas LTR_STRUC identified only 14 of them. The only missed known element, which is also missed by LTR_STRUC, is reported as two solo LTRs by our method. There are 6 common new elements identified by both methods. In contrast, each method identified a considerable number of new elements that are not identified by the other one (29 for our method and 48 for LTR_STRUC), indicating that these two methods may be complementary in identifying new LTR retroelements. Similar results are obtained when comparing both methods on the *D. melanogaster *genome. We constructed a reference set of putative intact retroelements in euchromatic regions based on the latest annotation in Flybase [9]. Since the intact and fragment elements are not distinguished in the annotation, we consider only 639 ones that are longer than 1000 bp as putative intact elements. Using only intact LTR retrotransposons in comparison, our method identified 418 elements in the reference set, whereas LTR_STRUCT identified 331 retroelements, and 320 of them are in the reference set (Figure 1(b)). When comparison was made using both intact and solo LTR retrotransposons, our method identified 569 elements in the reference set. These results show that our method has higher sensitivity than LTR_STRUC. The missed elements by our method may not contain any LTR.

#### LTR retrotransposons in the *C. elegans *genome

A total of 45 pairs of young intact LTRs were found in the *C. elegans *genome after step 1 (see Methods for details). The pairs of LTR sequences were then grouped into 37 clusters. A profile HMM was built for each cluster and subsequently searched against the whole genome and 323 solo LTRs were found at this step. Next we applied the phylogenetic approach to identify old intact LTR retrotransposons. 26 (out of 323) solo LTRs were paired at this step, which added 13 old intact LTR retrotransposons to the final result. In summary, we identified 58 intact LTR retrotransposons and 297 solo LTRs. The sequence identities between a pair of LTRs from intact retroelements were above 89.0% with an average value of 98.5%. The locations of these elements identified in the latest release (release 2) of the *C. elegans *genome sequence can be found in our complementary website. Previous studies based on homology searching have identified 24 intact LTR retrotransposons in 19 families from the *C. elegans *genome [14]. These families were defined according to the similarity of common protein domains such as reverse transcriptase (RT) or envelope protein (ENV). We successfully recovered 23 out of these 24 known intact LTR retrotransposons, and 17 out of 19 these known families (see Table 2). We note that our method is a *de novo *method that does not rely on a library of known elements. Therefore, the recovery of known retroelements is encouraging, indicating our method has a satisfactory false negative rate. Some of the previously known families correspond to one cluster in our findings, whereas in the other cases, two families are merged into a single cluster (i.e. *Cer*8/*Cer*9 and *Cer*12/*Cer*16). This shows that our automatic sequence clustering algorithm can define families of LTR retroelements similarly to the previous phylogenetic analyses. Nevertheless, through the rest of the paper, we still call the groups of LTR retroelements identified by our method *clusters *instead of *families*, implying that additional verification is required before some of them can be determined new LTR retrotransposon families. The only intact LTR retroelement that we missed is known to be an intact LTR retroelement of the family *Cer*20 (denoted as the cluster LTR_CE15 in our results) that does not have either common protein domains or long ORFs inside the element, which remains a hard case for any automatic identification method. Our program reported this pair of LTRs as two solo LTRs in cluster LTR_CE15, again because their sequences are not the most similar in the phylogenetic tree (see details on our complementary website). Two retrotransposon families that we missed (*Cer*11 and *Cer*14) do not contain intact elements (called solo-only families). We stress that as any other *de nov *method, our method focuses on the identification of intact elements and can identify solo elements only if there are intact elements from the same family present in the genome. Solo-only families, however, may be discovered through a homology-based method using LTR sequences identified from intact elements in other genomes (e.g., family *Cer*20).

Table 2 summarizes all clusters, including 22 new clusters, identified in this work. We note that these findings need to be validated by additional inspection. In addition to the new clusters, we also identified several new elements in some previously classified families [14]. For example, in our results, cluster LTR_CE8 is a mixture of two previously identified families (*Cer*8 and *Cer *9). Previous study identified, in total, 5 retroelements (2 and 3 respectively) in these two families, whereas our method identified 8 retroelements. One of the three new retroelements in this cluster (element 5) was identified in step 1 and the other two (element 6 and element 8) were identified in step 3. The similarities between the new elements and previously known elements in this cluster were significant, e.g. 45.6% (element 1 vs. 5), 44.3% (element 1 vs. 6) and 53.0% (element 2 vs. 8). We stress that all these new elements are not identified by LTR_STRUC in our test, indicating these missed elements are probably not caused by different versions of the *C. elegans *genome used in this study than in the previous one.

#### LTR retrotransposons in the *C. briggsae *vs. *C. elegans *genomes

**Figure 2:**
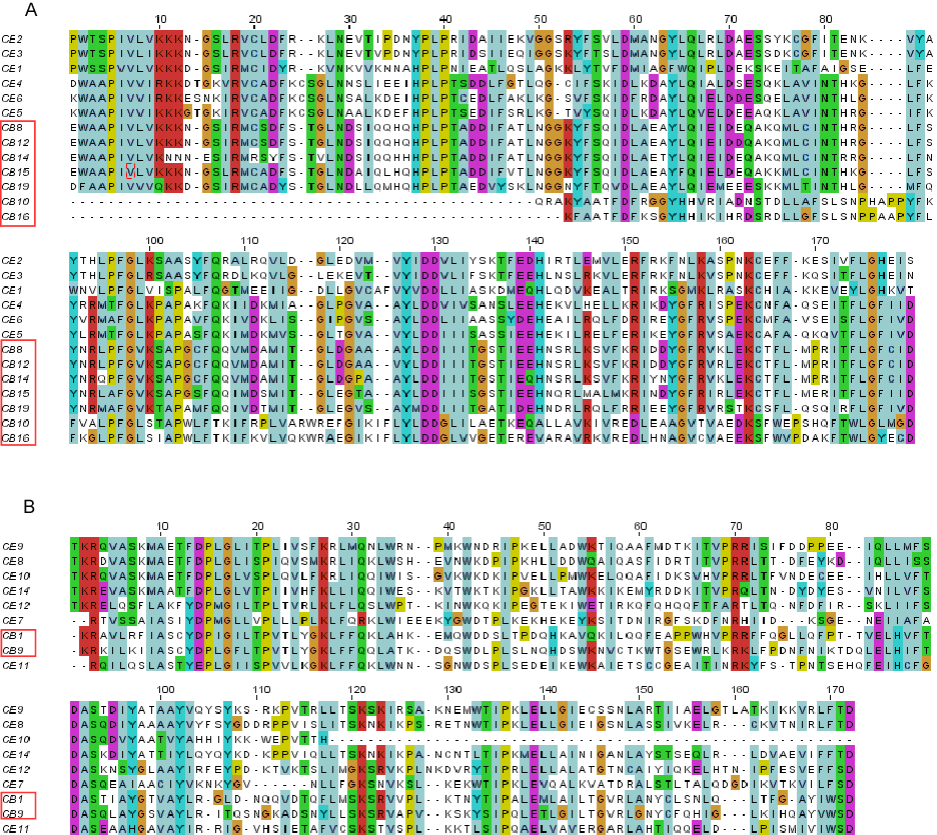
Alignment of RT domains in identified LTR retroelements from *C. briggsae *and *C. elegans*. (A) Domains from elements in LTR_CB8, 12, 14, 15, and 19 from *C. briggsae *(in red box) and *Cer *elements in *gypsy*/*Ty3 *group from *C. elegans*. (B) Domains from elements in LTR_CB1 and 9 from *C. briggsae *(in red box) and *Cer *elements in *Bel *group.

A total of 24 pairs of intact LTRs were found in the *C. briggsae *genome after step 1. The LTRs were clustered into 19 clusters, from which pHMM searching identified 277 solo LTRs. Using phylogenetic analyses in step 3, two additional intact LTRs were redefined from 4 (out of 277) solo LTRs. Hence, in total, our method identified 26 intact elements and 273 solo LTRs (see Additional file 1). We emphasize that we identified more elements than the conventional method in the *C. briggsae *genome. The UCSC genome browser[23] has annotated the MGEs for the *C. briggsae *genome based on RepeatMasker searching against updated Repbase. There are only 9 fragmentary sequences from the previously known LTR retrotransposon family (*Cer*1). However, all of them are shorter than the half-length of the known intact elements in this family. This result is also consistent with a similar cross-species analysis that we performed. When we used RepeatMasker to annotate retroelements in *C. briggsae *based on a library of LTR elements in *C. elegans*, we got 74 hits, but most of them were short partial matching and no significant hit was found. This suggests that the conventional homology searching methods may fail to identify new LTR retroelements, even when a library of LTR retroelements from a closely related organism is used. In contrast, our method has identified many more elements, including several putative new families, again demonstrating the advantages of our *de novo *method. Among these new elements, the largest cluster (LTR_CB16) contains five intact elements, in which the average identity between LTRs in the elements is above 98%. Three other clusters contain two intact elements and the remaining clusters contain only one intact element. The predicted RT domains in retroelements from the *C. briggsae *genome were aligned with those from the *C. elegans *genome in order to verify these identified elements (Figure 2). Based on the groups by phylogenetic analysis (described below), seven RT domains from clusters LTR_CB8, 10, 12, 14, 15, 16, and 19 in the *C. briggsae *genome were aligned along with RT domains from the known elements, LTR_CE1, 2, 3, 4, 5, and 6, in the *C. elegans *genome (Figure 2(a)). RT domain from two retroelements, LTR_CB1 and LTR_CB9, in the *C. briggsae *genome were aligned along with the known elements, LTR_CE7, 8, 9, 10, 11, 12, 13, and 14, in the *C. elegans *genome, which also belong to the same group in the phylogenetic tree (see Figure 2(b)). The aligned RT sequences from both genomes are well conserved, in particular in those OSM regions, implying that these elements identified by our methods are likely true LTR retroelements.

**Figure 3:**
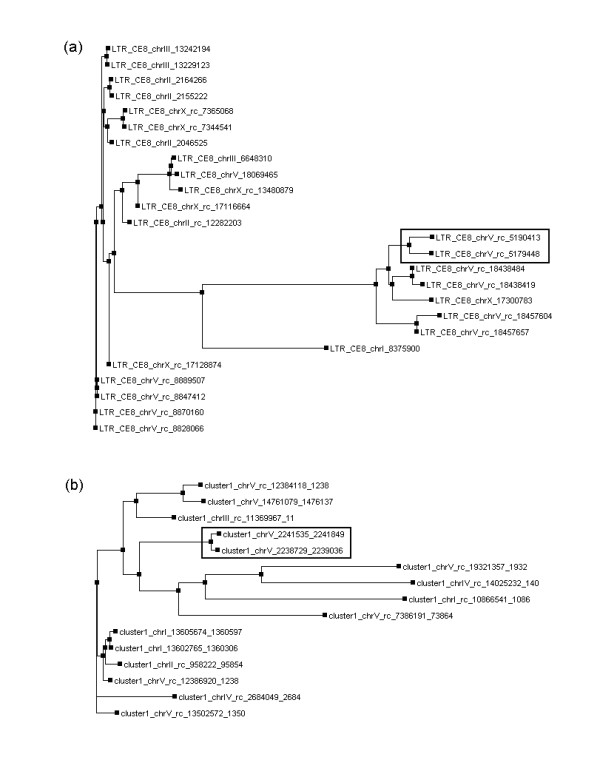
Phylogenetic trees of clusters LTR_CE8 (a) and LTR_CE15 (b) in *C. elegans*. The elements LTR_CE8_chrV_rc_5190413 and LTR_CE8_chrV_rc_5179448 in tree (a) and LTR_CE15_chrV_2241535 and LTR_CE15_chrV_2238729 in tree (b), highlighted in rectangular boxes, are intact LTR retroelements identified by phylogenetic analysis.

The sequence-based LTR finding method, as described previously [24] or used in step 1 in our program, may miss some *old *retroelements, i.e. those elements containing pairs of LTRs with lower identities. Our phylogenetic analysis (step 3) may overcome this disadvantage and identify additional intact retroelements. For example, the intact LTR retroelements (full-7) in cluster LTR_CE8 from *C. elegans *(see our companion website), which was also identified by an homology searching method in previous studies [14], was missed by step 1 in our method. However, this intact LTR was identified in step 3 of our program by phylogenetic analysis, because in this case the pair of LTRs was the closest neighbors on the phylogenetic tree of the entire LTR family (Figure 3(a), highlighted in gray box). Another example is an intact LTR retroelement (full-2) in cluster LTR_CE15 in the *C. elegans *genome (see our complementary website). In this cluster, one intact element (full-1) was identified in step 1, whereas the other one (full-2) was missed in step 1, but identified in step 3 since its pair of LTRs were the closest in the phylogenetic tree of the entire LTR family (Figure 3(b), highlighted in grey box).

We note that the number of elements identified in the *C. briggsae *genome is significantly less than in the *C. elegans *genome, even though these two genomes have similar genome size and gene content [25]. We hypothesize that this difference may be due to the fact that the *C. briggsae *genome is not fully finished. We used the following simulation experiment to test this hypothesis. We randomly shredded the sequence of the *C. elegans *genome into the same number (577) of scaffolds with identical lengths to those of the *C. briggsae *genome. We repeated this procedure 100 times and each time we determined how many identified intact elements in the *C elegans *genome were broken. We found that on average 31 (out of 58) intact elements were retained (with standard deviation about 9), which is comparable to the number of intact elements identified in the *C. briggsae *genome (24). Hence, we concluded that the *C. briggsae *genome may not contain significantly fewer intact LTR retroelements than the *C. elegans *genome, and that many elements may still be missing from the current analysis because of the incompleteness of its genomic sequence.

#### LTR retrotransposons in *D. melanogaster *genome

A total of 601 pairs of LTRs from young intact retroelements were identified in the *D. melanogaster *genome after step 1. The pairs of LTR sequences were grouped into 113 clusters. Searching against the whole genome based on the profile HMM of intact LTRs for each cluster, we identified 5595 solo LTRs. In step 3, the phylogenetic approach was applied and identified 85 old intact LTR retroelements from 170 (out of 5595) solo LTRs. Hence, in summary, we identified 686 (= 601+85) intact LTRs and 5425 (= 5595-170) solo LTRs. These LTR retroelements were compared with previously identified families. A total of 22 LTR retrotransposon families in common were reported in four independent studies [15,26-29]. Among 113 clusters that we identified, 22 clusters are equivalent to these 22 families. Table 3 summarizes these 22 clusters together with some other clusters containing more than four intact retroelements. The cluster LTR_DM17 contains the largest number (98) of intact LTR elements. The average identity of LTR pairs from intact retroelements was 99.7%. The cluster LTR_DM2 (equivalent to the known family 17.6 [15,26]), contains 16 intact LTR retroelements. The identities between 15 (out of 16) LTR pairs are above 99% and the average is 99.5%.

#### LTR retrotransposons in the *D. pseudoobscura vs. D. melanogaster *genomes

A total of 43 pairs of intact LTR retrotransposons were identified after step 1 in the *D. pseudoobscura *genome. The LTRs thus obtained were clustered into 41 clusters, from which 983 solo LTRs were found. After phylogenetic analysis, 5 additional old intact LTR retroelements were identified from 10 (out of 983) solo LTRs. In summary, we identified 48 (= 43+5) intact retrotransposons and 973 (= 983-10) solo LTRs (see Additional file 2). We identified far fewer LTR retroelements in the *D. pseudoobscura *genome than in the *D. melanogaster *genome. This is also understandable since the *D. pseudoobscura *genome is not as well finished as the *D. melanogaster *genome. In particular, almost no heterochromatic DNA has been sequenced in this genome. In contrast, there is a well-progressed finishing effort for the *D. melanogaster *genome, particularly in heterochromatic regions. As we show below, in the *D. melanogaster *genome, a major fraction of the LTR retroelements were identified in heterochromatic regions. Nevertheless, we can still identify many putative LTR retroelements in euchromatic regions of the *D. pseudoobscura *genome. For example, in cluster LTR_DP30, two intact LTR retroelements were identified in step 1. The identities between the pairs of LTRs are 96.3% and 98.0%, respectively. The identity between these two elements is 62.6%.

### Chromosomal distribution of LTR retroelements

The analysis of chromosomal distributions of the identified LTR retroelements was performed on the *C. elegans *and *D. melanogaster *genomes. The Kolmogorov-Smirnov test was used to determine whether the LTRs are distributed uniformly in terms of their chromosomal location. With the significance level of 0.05, the hypothesis of a uniform distribution was clearly rejected in chromosomes I, II, V, and X of the *C. elegans *genome (see Additional file 3); whereas the significance level on chromosomes III and IV were p = 0.4586 and p = 0.1420, respectively. These results were consistent with the previous observations on the same genome [14] and the DNA replication model for the *C. elegans *genome [30].

**Figure 4:**
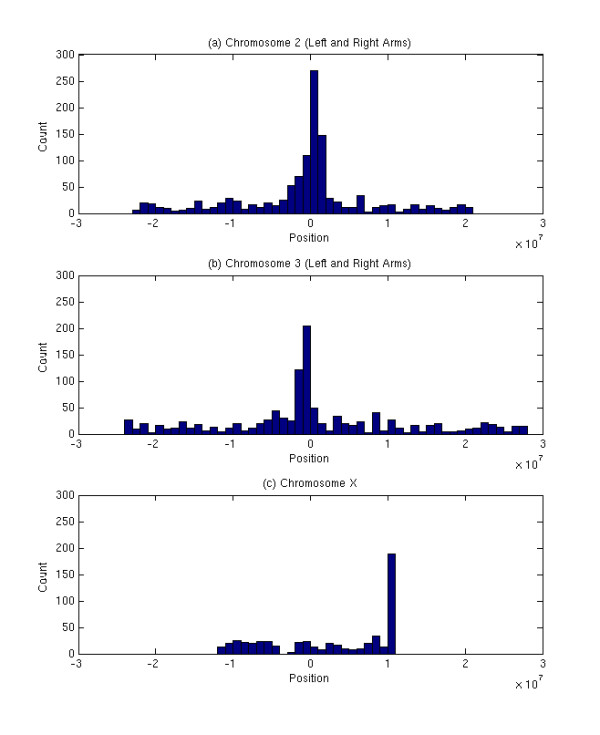
Density of LTRs as a function of chromosomal position in chromosome II (a), chromosome III (b), and chromosome X (c) of *D. melanogaster*. The left sides of 0 in (a) and (b) are from left arms and the right sides of 0 in (a) and (b) are from right arms. The middled point of X chromosome is defined as 0 for the comparison. High densities of LTRs are observed in the pericentromeric regions of all three chromosomes.

The same test was performed for four chromosomes (chromosome 2, 3, 4, and X) in the *D. melanogaster *genome. The results showed that the hypothesis was rejected in all four chromosomes. As shown in Figure 4, LTRs are more densely distributed in the pericentromeric regions of the chromosomes 2 and 3 (at the end of the chromosome arms 2L and 3L and at the beginning of the chromosome arms 2R and 3R) which are also considered to be highly heterochromatic regions. The chromosome X has more LTRs on one end. It was determined that 47% and 39% of the LTRs (intact elements and solos) are located in the proximal 6 Mb of the pericentromeric regions of chromosomes 2 and 3, respectively. Similarly, 44% of the LTRs are located in the proximal 3 Mb of the pericentromeric regions of chromosome X. In order to see whether LTR retroelements are distributed uniformly in euchromatic regions, the test was carried out with the same hypothesis after excluding 6 Mb of the pericentromeric regions of chromosomes 2 and 3. The result showed that the hypothesis was still rejected in these two chromosomes, indicating that there might be regions for more LTR retroelement insertions even in euchromatic regions.

### Phylogenetic analysis of RT domains

**Figure 5:**
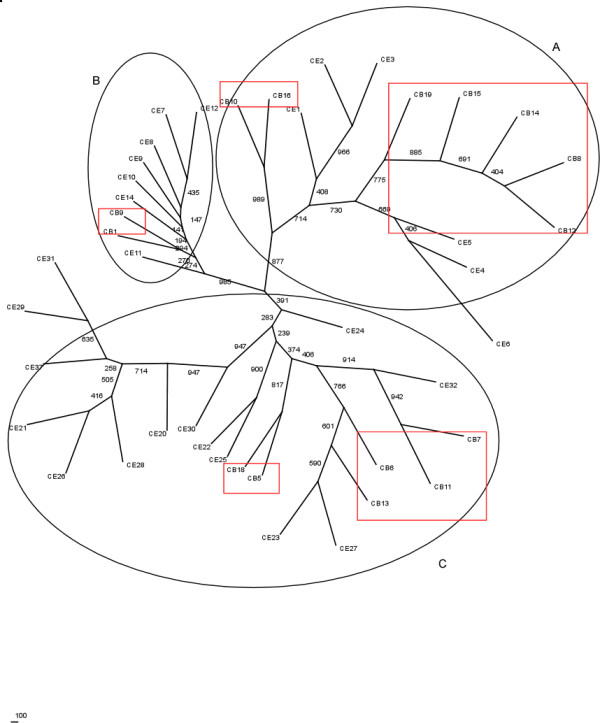
The neighbor-joining phylogenetic tree of RT domains in LTR retroelements from the *C. elegans *(CE) and the *C. briggsae *(CB) genomes. Bootstraps support values are indicated. New elements from the *C. briggsae *genome are boxed in red.

We used the RT domains in LTR retroelements to analyze the phylogenetic relationships of the LTR retroelements from two close species, *C. elegans *and *C. briggsae*. The elements that were identified by our method can be divided into two groups. One group has a known RT domain whereas the other one has long ORFs (>700 bp) that may represent novel RT domains. From the elements identified in the first group of the *C. elegans *and the *C. briggsae *genomes, the RT protein sequences were extracted. Their neighbor-joining phylogenetic tree with 1000 bootstraps was generated (Figure 5). The tree contains three major groups, of which two groups contain known elements. Group A in Figure 5 contains elements in clusters LTR_CE1-6, which are compatible with *Cer *1–7 in *gypsy/Ty1 *group from the previous study [14] and the new elements in clusters LTR_CB8, 10, 12, 14, 15, 16, and 19 of the *C. briggsae *genome. Group B in Figure 5 contains elements in clusters LTR_CE 7–14, which are compatible with *Cer *8–20 in *Bel *group from the previous study [14] and the new elements in clusters LTR_CB1 and 9 of the *C. briggsae *genome. The third group C contains new elements in the *C. elegans *genome and *C. briggsae *genome. We note that this preliminary analysis needs to be analyzed further based on retroelements identified in multiple genomes.

**Figure 6:**
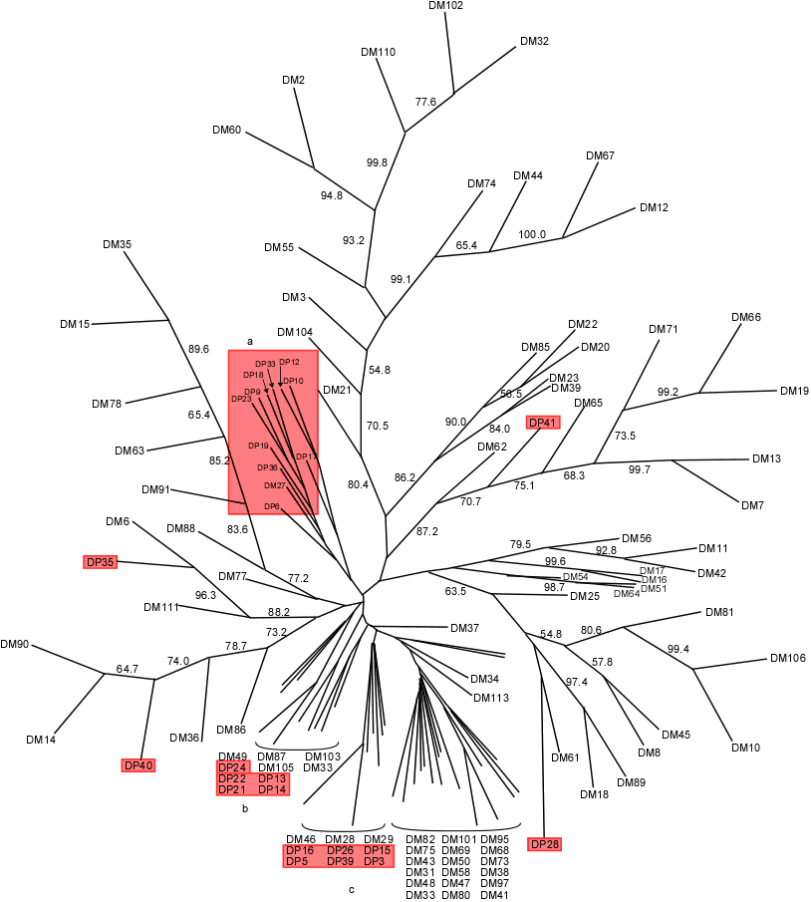
The neighbor-joining phylogenetic tree of reverse transcriptase (RT) domains in LTR retroelements from the *D. melanogaster *(DM) and the *D. pseudoobscura *(DP) genomes. Bootstrap support (> 50%) is indicated. Clusters from the *D. pseudoobscura *genome are highlighted in red.

A neighbor-joining phylogenetic tree was also generated for the RT domains identified by our method in LTR retroelements from *D. melanogaster *and *D. pseudoobscura *(Figure 6). Interestingly, the bootstrap support value is greater than 50% in most of the branches except those in the bottom. The clusters (DP28, DP35, DP40, and DP41, highlighted in red) are located in the group of clusters from *D. melanogaster*. One group (a) contains clusters only from the *D. pseudoobscura *genome while two other groups (b and c) are mixed of clusters from both genomes.

## Conclusion

We proposed a novel computational method for *de novo *identification of LTR retrotransposons in eukaryotic genomes. It has been applied to several complete eukaryotic genomes and identified many new putative intact LTR retroelements, among which a few new potential families were discovered.

## Methods

**Figure 7:**
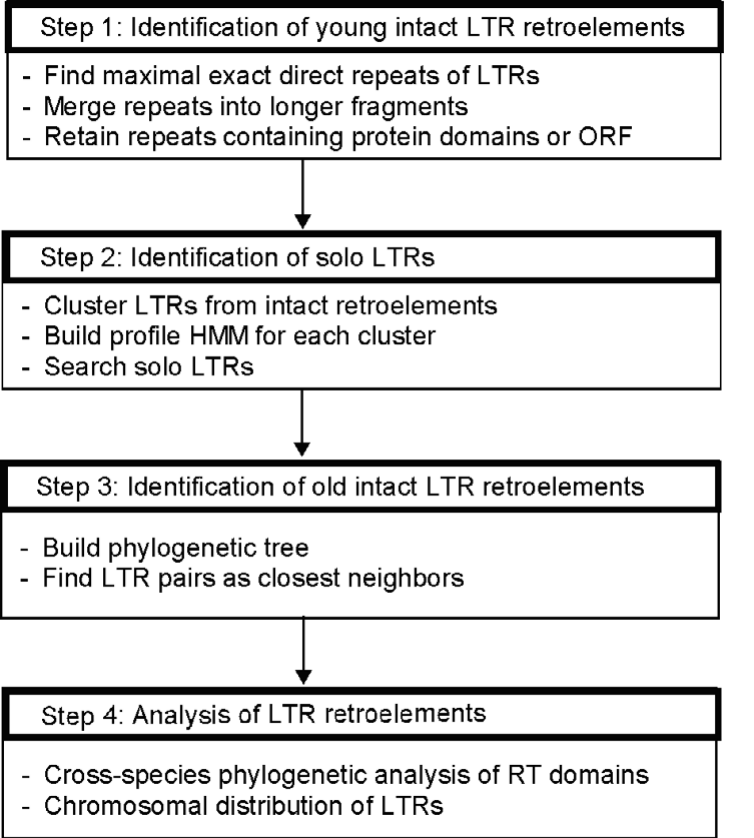
The pipeline of LTR retrotransposon identification used in this paper.

Our *de novo *approach to identifying LTR retrotransposons consists of three steps, each using different algorithms (Figure 7): (1) identification of young intact LTR retrotransposons; (2) identification of solo LTRs; (3) identification of old intact LTR retrotransposons. We also developed several computer programs to analyze the identified LTR retrotransposons.

### Genomic sequences

The genomic sequences of *C. elegans*, *C. briggsae*, *D. melanogaster*, and *D. pseudoobscura *were obtained from public domains. The complete genomic sequence of *C. elegans *(WS120) and a draft genomic sequence of *C. briggsae *(cb25.agp8) were downloaded from Wormbase at the Sanger Institute[31]. The complete genomic sequence of *D. melanogaster *(Release 4.0) was downloaded from the website of the Berkeley Drosophila Genome Project [32]. The draft genomic sequence of *D. pseudoobscura *(Release 1.0) was downloaded from FlyBase [33].

### *De novo *identification of young intact LTR retroelements

**Figure 8:**
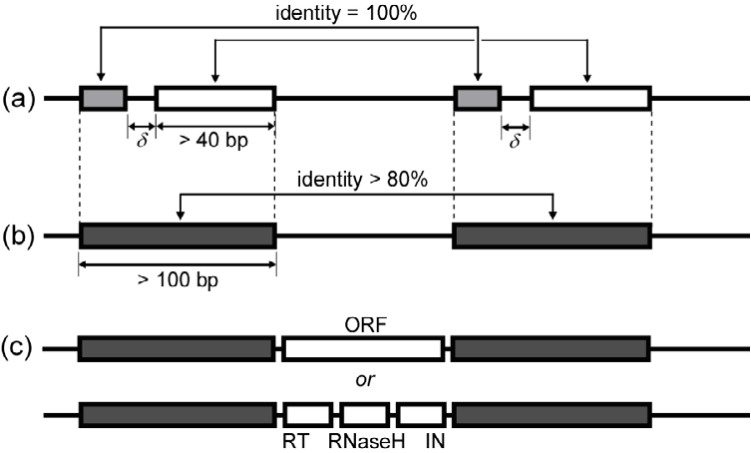
Identification of intact LTR retroelements (Step 1 in Figure 7) consists of three steps. (a) Pairs of maximal exact direct repeats (> 40 bp) were found; (b) Repeats were merged if they were proximal in the genome locations, if the length of the merged repeats is greater than 100 bp and the identity is greater than 80%; (c) If the segment capped by repeats contain frequent protein domains (RT, RNaseH, or IN), or an ORF (>700 bp), they were retained.

Given a genomic sequence, we first try to identify young intact LTR retrotransposons. Each intact LTR retroelement contains a pair of LTRs at each end (5' and 3'). It is generally known that the length of LTRs ranges from 100 to 1000 bp and their distance (including the length of the two LTRs, or the entire length of the intact element) ranges from 1000 to 20000 bp. The age of intact LTR retrotransposons may be dated by the identity between their two LTRs, because these two LTRs are identical at the time of transposition. Many of the intact LTR retroelements are young, i.e. they were transposed into their current locations in recent evolutionary history, and hence, the identities between their LTRs are high. Our approach to finding these young intact LTR retroelements is equivalent to finding pairs of highly similar short subsequences (LTRs, between 100 and 1000 bps long) located within a range of distance (between 1000 to 20000) from the given genome sequence. We adopted a fast approximate string matching algorithm similar to that previously reported [24]. The entire procedure consists of three heuristic steps (Figure 8). The first step is to find pairs of *maximal exact *direct repeats that are longer than 40 bp and located within a range of distances (between 1000 bp and 20000 bp). This step can be done in linear time using a suffix array data structure [34]. We modified a module of GAME [35], which rapidly aligned microbial genomic sequences based on MEM (Maximal Exact Match) detection using suffix array and bottom-up traversal of suffix trees [36]. While traversing in a bottom-up fashion, each node in the suffix array utilizes a hash structure to map a character to a position list, which indexes all substrings and their leftmost characters. When visiting a leaf node, the corresponding suffix string is added to the position list of its leftmost character. MEMs then can be detected by a self cross-product of the array. In the second step, these (short) exact direct repeats were merged into longer fragments by combining multiple direct repeats if two consecutive repeats are in close proximity with intervening lengths less than 20 bp. Pairs of merged fragment (potential pairs of LTRs) within a range of lengths (between 100 and 2000 bp) and with identities greater than 80% were retained. We stress that using the criteria described above, we can only identify those pairs of subsequences (fragments) that are very similar to each other (i.e. containing at least a 40 bp long identical subsequences and with an overall identity higher than 80%). As a result, we may miss some relatively old intact LTR retroelements, of which some can be recovered by the next steps of our methods. In the third step, we scan open reading frames (ORFs) within the sequence in the middle of each pair of fragments (potential LTR retroelements) using Hidden Markov Models (HMMs) of protein domains that are often observed in LTR retrotransposons, including group-specific antigen (gag), protease (prt), reverse transcriptase (RT), RNaseH, and integrase (IN), all taken from Pfam database (version 19) [37]. The scan was conducted using HMMSearch from profile HMM package HMMER, obtained from Washington University [38]. We retained only those pairs of fragments containing a set of protein domains having a combined E-value less than a threshold (1.0e-10); or containing a long enough ORF (> 700 bp). We retained the candidate LTR retroelements containing no known frequent protein domains, but with a long ORF, to avoid missing completely new elements. In the last step, we eliminated those pairs of fragments (potential LTRs) matching with known repeats defined as DNA transposons in Repbase [3], which are likely false positives (i.e. two transposons that were inserted into proximal locations instead of a single LTR retrotransposon). The locations of these RNA transposons in these four genomes were obtained from UCSC Genome Browser [23].

### Identification of Solo LTRs

Solo LTRs are created by recombination between two intact LTRs during evolution. In order to identify solo LTRs, LTRs from intact retroelements identified in the previous step were first clustered based on their sequence similarity, using a BAG clustering algorithm [22]. The BAG clustering algorithm represented all LTRs from intact retroelements by an undirected graph, in which each node represented a LTR sequence and a weighted edge between two LTR were created if the sequence similarity between corresponding LTRs was above a preset cutoff threshold. Smith-Waterman alignment score from FASTA comparison of two LTRs was used as a similarity measure. BAG generated clusters of LTRs by iteratively splitting a graph into biconnected components with an increased cutoff score at each iteration while forcing two LTRs from the same intact element to be grouped into the same cluster. Next, for each cluster of LTR sequences, we aligned them using CLUSTALW and the resulting multiple alignment was used to generate a profile HMM using HMMBuild from the HMMER package. Finally, HMMSearch from the same package was used to search for HMMs from all the LTR clusters against the entire genome to identify potential LTRs, including solos. The threshold of E-value for the search was set up as 1.0e-9, which was determined based on the best recovery of known solo LTRs.

### Identification of old intact LTR retroelements

In the previous sections, we have shown how we identified young LTR retroelements that contain highly similar pairs of LTRs. However, this approach may miss those relatively old LTR retrotransposons that contain two LTRs that are no longer highly similar to each other. To address this issue, a phylogenetic analysis was carried out. We built a phylogenetic tree for all solo LTRs in the same cluster. Some LTRs among them may not be true solos; instead they may be located within certain distance ranges and the sequence between them may actually be an intact retroelement. The reason why they are classified as "solo LTRs" is simply because they are not highly similar to each other to be identified based on the criteria used in the first step. We classified a pair of "solo" LTRs into a single (old) intact retroelement, if they are (1) located within certain distance range in the genome; and (2) closest neighbors in the phylogenetic tree (Figure 1).

### Software implementation

We implemented the method described above in a software package, using C++ and Perl. The source code of the major part of program can be downloaded from the supplementary website [39]. The typical running time for analyzing a eukaryotic genome ranges from several hours to tens of hours.

### Phylogenetic analysis

Throughout the paper, all phylogenetic analysis was done in two steps. The sequences were first aligned using CLUSTALW [40] and then the neighbor-joining tree was built using PHYLIP [41] with 1000 bootstraps.

### Analysis of the distribution of the genomic locations of LTR retroelements

The chromosomal distribution of LTRs was analyzed by a Kolmogorov-Smirnov (KS) test. The null hypothesis based on a uniform distribution was used to determine whether the chromosomal distribution of LTRs is random. The pre-defined function for KS test in MATLAB was used for this purpose. For further analyses, the chromosomal distribution of the ratios between the number of intact LTR retroelements and solo LTRs in the *D. melanogaster *genome was computed and plotted along coordinate bins of chromosomes 2, 3, and X.

## Authors' contributions

HT, ML and SK conceived of the study, and participated in its design and coordination. MR, JC, SK and HT carried out the computational analysis. MR, SK and HT drafted the paper. All authors read and approved the final manuscript.

## Supplementary Material

Additional File 1 List of LTR retroelements in the *C. briggsae *genome. Click here for file

Additional File 2 List of LTR retroelements in the *D. pseudoobscura *genome. Click here for file

Additional File 3 Distribution of LTR retroelements in the *C. elegans *genome. The coordinates of elements are plotted with respect to their chromosomal locations. Click here for file

## Figures and Tables

**Table 1:** Number of Clusters, Intact LTRs and Solo LTRs in the genomes of *C. elegans*, *C. briggsae*, *D. melanogaster*, and *D. pseudoobscura*

Genome	Clusters	Intact LTRs	Solo LTRs
*C. elegans*	37	58	297
*C. briggsae*	19	26	273
*D. melanogaster*	113	686	5425
*D. pseudoobscura*	41	48	973

**Table 2:** List of Elements in the *C. elegans *genome

Cluster	Family notation from previous work	# of Intact LTRs (from previous work)	Avg. Identity between LTRs (%)	# of Solo LTRs (from previous work)
LTR_CE1	Cer1	1(1)	99.8	3(3)
LTR_CE2	Cer2	1(1)	99.2	5(4)
LTR_CE3	Cer3	2(1)	99.8	12(11)
LTR_CE4	Cer4	1(1)	99.3	0(0)
LTR_CE5	Cer5	2(1)	98.95	13(13)
LTR_CE6	Cer6	1(1)	99.1	7(5)
LTR_CE7	Cer7	1(1)	99.7	1(1)
LTR_CE8	Cer8/Cer9	8(2/3)	98.25	8(1/8)
LTR_CE9	Cer10	1(1)	99.5	5(4)
LTR_CE10	Cer12/Cer16	6(1/2)	98.2	28(14/13)
LTR_CE11	Cer13	1(1)	98.8	2(1)
LTR_CE12	Cer15	3(2)	98.8	4(5)
LTR_CE13	Cer17	1(1)	99.7	0(0)
LTR_CE14	Cer19	2(1)	99.75	8(10)
LTR_CE15	Cer20	2(3)	97.6	9(5)
LTR_CE16		1	96.4	0
LTR_CE17		3	84.23	17
LTR_CE18		4	96.75	21
LTR_CE19		1	97.8	2
LTR_CE20		1	99.0	45
LTR_CE21		1	98.8	0
LTR_CE22		1	99.4	2
LTR_CE23		3	92.9	27
LTR_CE24		1	98.3	0
LTR_CE25		1	97.2	0
LTR_CE26		1	97.2	0
LTR_CE27		1	99.3	2
LTR_CE28		1	99.5	0
LTR_CE29		1	99.2	22
LTR_CE30		2	86.5	0
LTR_CE31		1	99.3	0
LTR_CE32		1	99.1	0
LTR_CE33		2	98.6	1
LTR_CE34		6	96.88	42
LTR_CE35		1	99.6	1
LTR_CE36		1	99.0	0
LTR_CE37		2	97.35	0

**Table 3:** List of Elements in the *D. melanogaster *genome

Cluster	Family notation from previous work	# of Intact LTRs (from previous work)	Avg. Identity between LTRs (%)	# of Solo LTRs
LTR_DM1	1731	4 (n/a, 1)	99.3	15
LTR_DM2	17.6	16 (6, 7)	99.5	29
LTR_DM3	297	38 (10, 18)	99.8	55
LTR_DM4	412	28 (18, 24)	99.8	16
LTR_DM5	bel	4 (n/a, 4)	99.6	13
LTR_DM6	blastopia	16 (6, 13)	100.0	4
LTR_DM7	blood	23 (21, 22)	99.8	16
LTR_DM8	burdock	13 (1, 7)	99.4	24
LTR_DM9	copia	30 (n/a, 26)	99.6	8
LTR_DM10	gypsy	8 (n/a, 1)	97.9	52
LTR_DM11	HMS beagle	17 (8, 9)	98.4	42
LTR_DM12	idefix	4 (n/a, 2)	98.7	69
LTR_DM13	mdg1	19 (4, 13)	99.9	23
LTR_DM14	mdg3	13 (8, 8)	99.2	16
LTR_DM15	micropia	3 (1, 2)	100.0	10
LTR_DM16	nomad	16 (4, 16)	99.9	23
LTR_DM17	roo	98 (40, 58)	99.7	96
LTR_DM18	springer	17 (n/a, 5)	99.3	75
LTR_DM19	stalker	25 (n/a, 3)	98.3	104
LTR_DM20	tirant	21 (6, 15)	99.8	32
LTR_DM21	transpac	5 (4, 5)	99.7	7
LTR_DM22	ZAM	3 (n/a, 0)	96.1	9
LTR_DM44		5	99.1	62
LTR_DM60		7	97.6	24
LTR_DM63		5	99.2	91
LTR_DM64		13	96.9	122
LTR_DM66		10	99.7	93
LTR_DM67		6	96.5	95
LTR_DM70		9	99.5	5
LTR_DM74		8	95.8	58
LTR_DM77		9	99.0	16
LTR_DM79		8	98.4	32
LTR_DM80		7	98.6	18
LTR_DM82		11	99.2	46
LTR_DM88		6	97.5	57
LTR_DM90		7	99.4	30
LTR_DM97		8	95.1	38
LTR_DM100		6	99.1	18
LTR_DM103		5	94.8	197
LTR_DM106		5	99.2	109
LTR_DM107		7	99.1	27
LTR_DM111		5	97.9	17

## References

[B1] Kidwell MG, Lisch D (1997). Transposable elements as sources of variation in animals and plants.. Proc Natl Acad Sci U S A.

[B2] Brookfield JF (2005). The ecology of the genome - mobile DNA elements and their hosts.. Nat Rev Genet.

[B3] Jurka J (2000). Repbase update: a database and an electronic journal of repetitive elements.. Trends Genet.

[B4] Smit A RepeatMasker. http://www.genome.washington.edu/uwgc/analysistools/repeatmask.htm.

[B5] Holmes I (2002). Transcendent elements: whole-genome transposon screens and open evolutionary questions.. Genome Res.

[B6] Bao Z, Eddy SR (2002). Automated de novo identification of repeat sequence families in sequenced genomes.. Genome Res.

[B7] Price AL, Jones NC, Pevzner PA (2005). De novo identification of repeat families in large genomes.. Bioinformatics.

[B8] Edgar RC, Myers EW (2005). PILER: identification and classification of genomic repeats.. Bioinformatics.

[B9] Quesneville H, Bergman CM, Andrieu O, Autard D, Nouaud D, Ashburner M, Anxolabehere D (2005). Combined evidence annotation of transposable elements in genome sequences.. PLoS Comput Biol.

[B10] Bailey JA, Eichler EE (2003). Genome-wide detection and analysis of recent segmental duplications within mammalian organisms.. Cold Spring Harb Symp Quant Biol.

[B11] Jiang N, Bao Z, Zhang X, Hirochika H, Eddy SR, McCouch SR, Wessler SR (2003). An active DNA transposon family in rice.. Nature.

[B12] Caspi A, Pachter L (2006). Identification of transposable elements using multiple alignments of related genomes. Genome Research.

[B13] SanMiguel P, Gaut BS, Tikhonov A, Nakajima Y, Bennetzen JL (1998). The paleontology of intergene retrotransposons of maize.. Nat Genet.

[B14] Ganko EW, Fielman KT, McDonald JF (2001). Evolutionary History of Cer Elements and Their Impact on the C. elegans Genome. Genome Research.

[B15] Kaminker JS, Bergman CM, Kronmiller B, Carlson J, Svirskas R, Patel S, Frise E, Wheeler DA, Lewis SE, Rubin GM, Ashburner M, Celniker SE (2002). The transposable elements of the Drosophila melanogaster euchromatin: a genomics perspective. Genome Biology.

[B16] Lerat E, Rizzon C, Biémont C (2003). Sequence Divergence Within Transposable Element Families in the Drosophila melanogaster Genome. Genome Research.

[B17] McCarthy EM, McDonald JF (2004). Long terminal repeat retrotransposons of Mus musculus.. Genome Biol.

[B18] Ma J, Devos KM, Bennetzen JL (2004). Analyses of LTR-Retrotransposon Structures Reveal Recent and Rapid Genomic DNA Loss in Rice. Genome Research.

[B19] McCarthy EM, McDonald JF (2003). LTR_STRUC: a novel search and identification program for LTR retrotransposons.. Bioinformatics.

[B20] Kalyanaraman A, Aluru S (2006). Efficient algorithms and software for detection of full-length LTR retrotransposons. J Bioinform Comput Biol.

[B21] Havecker ER, Gao X, Voytas DF (2004). The diversity of LTR retrotransposons.. Genome Biol.

[B22] Kim S, Lee J (2006). A Graph Theoretic Sequence Clustering Algorithm. Int J Data Mining Bioinformatics.

[B23] http://genome.ucsc.edu.

[B24] Kalyanaraman A, Aluru S, Markstein P, Xu Y (2005). Efficient Algorithms and Software for Detection of Fell-Length LTR Retrotransposons: Stanford University..

[B25] Stein LD, Bao Z, Blasiar D, Blumenthal T, Brent MR, Chen N, Chinwalla A, Clarke L, Clee C, Coghlan A, Coulson A, D'Eustachio P, Fitch DH, Fulton LA, Fulton RE, Griffiths-Jones S, Harris TW, Hillier LW, Kamath R, Kuwabara PE, Mardis ER, Marra MA, Miner TL, Minx P, Mullikin JC, Plumb RW, Rogers J, Schein JE, Sohrmann M, Spieth J, Stajich JE, Wei C, Willey D, Wilson RK, Durbin R, Waterston RH (2003). The genome sequence of Caenorhabditis briggsae: a platform for comparative genomics.. PLoS Biol.

[B26] Bowen NJ, McDonald JF (2001). Drosophila Euchromatic LTR Retrotransposons are Much Younger Than the Host Species in Which They Reside. Genome Research.

[B27] Kapitonov VV, Jurka J (2003). Molecular paleontology of transposable elements in the Drosophila melanogaster genome. Proc Natl Acad Sci USA.

[B28] Rizzon C, Marais G, Gouy M, Biémont C (2002). Recombination Rate and the Distribution of Transposable Elements in the Drosophila melanogaster Genome. Genome Research.

[B29] Vieira C, Lepetit D, Dumont S, Biémont C (1999). Wake Up of Transposable Elements Following Drosophila simulans Worldwide Colonization. Mol Biol Evol.

[B30] Riddle DL, Blumenthal T, Meyse BJ, Priess JR (1997). C. elegans II.

[B31] ftp://ftp.sanger.ac.uk/pub/wormbase.

[B32] http://www.fruitfly.org/sequence/release4genomic.shtml.

[B33] Flybase ftp://ftp.hgsc.bcm.tmc.edu/pub/data/Dpseudoobscura.

[B34] Kasai T, Lee G, Arimura H, Arikawa S, Park K (2002). Linear-time longest common-prefix computation in suffix arrays and its applications.: Jerusalem, Israel..

[B35] Choi JH, Cho HG, Kim S (2005). Alignment method for microbial whole Genomes using maximal exact match filtering. Computational Biology and Chemistry.

[B36] Manber U, Myers G (1993). Suffix arrays: a new method for on-line string searches. SIAM J Comput.

[B37] Bateman A, Coin L, Durbin R, Finn RD, Hollich V, Griffiths-Jones S, Khanna A, Marshall M, Moxon S, Sonnhammer EL, Studholme DJ, Yeats C, Eddy SR (2004). The Pfam protein families database.. Nucleic Acids Res.

[B38] HMMer http://hmmer.wustl.edu.

[B39] http://darwin.informatics.indiana.edu/cgi-bin/evolution/ltr.pl.

[B40] Ramu C, Sugawara H, Koike T, Lopez R, Gibson TJ, Higgins DG, Thompson JD (2003). Multiple sequence alignment with the Clustal series of programs. Nucleic Acids Res.

[B41] Felsenstein J (1989). PHYLIP - Phylogeny Inference Package (Version 3.2). Cladistics.

