# Supplementary material for: De novo identification of LTR retrotransposons in eukaryotic genomes
Source: BMC Genomics. 2007 Apr 3;8:90. doi: 10.1186/1471-2164-8-90 (PMC1858694; doi:10.1186/1471-2164-8-90)
Supplement: Additional File 1 — List of LTR retroelements in the C. briggsae genome. [file 1471-2164-8-90-S1.doc]

**Supplementary Table 1. List of LTR retroelements in the *C. briggsae* genome**

| Cluster | # of Intact LTRs | Avg. Identity between LTRs (%) | # of Solo LTRs (from previous work) |
| --- | --- | --- | --- |
| LTR_CB1 | 1 | 99.6 | 5 |
| LTR_CB2 | 1 | 97.6 | 0 |
| LTR_CB3 | 1 | 99.1 | 3 |
| LTR_CB4 | 1 | 99.5 | 5 |
| LTR_CB5 | 1 | 98.7 | 76 |
| LTR_CB6 | 1 | 99.8 | 12 |
| LTR_CB7 | 1 | 98.1 | 32 |
| LTR_CB8 | 2 | 99.5 | 7 |
| LTR_CB9 | 1 | 100.0 | 1 |
| LTR_CB10 | 1 | 99.5 | 3 |
| LTR_CB11 | 1 | 98.5 | 5 |
| LTR_CB12 | 1 | 98.1 | 1 |
| LTR_CB13 | 2 | 88.25 | 11 |
| LTR_CB14 | 1 | 99.6 | 1 |
| LTR_CB15 | 1 | 98.2 | 23 |
| LTR_CB16 | 5 | 99.0 | 28 |
| LTR_CB17 | 1 | 98.1 | 0 |
| LTR_CB18 | 1 | 97.7 | 51 |
| LTR_CB19 | 2 | 92.8 | 8 |
