# Supplementary material for: De novo identification of LTR retrotransposons in eukaryotic genomes
Source: BMC Genomics. 2007 Apr 3;8:90. doi: 10.1186/1471-2164-8-90 (PMC1858694; doi:10.1186/1471-2164-8-90)
Supplement: Additional File 2 — List of LTR retroelements in the D. pseudoobscura genome. [file 1471-2164-8-90-S2.doc]

**Supplementary Table 2. List of LTR retroelements in the *D. pseudoobscura* genome**

| Cluster | # of Intact LTRs | Avg. Identity between LTRs (%) | # of Solo LTRs |
| --- | --- | --- | --- |
| LTR_DP1 | 1 | 98.8 | 9 |
| LTR_DP2 | 1 | 99.2 | 1 |
| LTR_DP3 | 1 | 97.9 | 59 |
| LTR_DP4 | 1 | 99.3 | 3 |
| LTR_DP5 | 1 | 98.1 | 60 |
| LTR_DP6 | 1 | 96.4 | 19 |
| LTR_DP7 | 1 | 99.1 | 68 |
| LTR_DP8 | 2 | 97.4 | 14 |
| LTR_DP9 | 1 | 98.2 | 9 |
| LTR_DP10 | 2 | 93.4 | 36 |
| LTR_DP11 | 1 | 98.9 | 1 |
| LTR_DP12 | 1 | 97.3 | 17 |
| LTR_DP13 | 2 | 97.1 | 37 |
| LTR_DP14 | 1 | 99.6 | 3 |
| LTR_DP15 | 1 | 98.6 | 36 |
| LTR_DP16 | 5 | 99.2 | 68 |
| LTR_DP17 | 2 | 98.1 | 24 |
| LTR_DP18 | 1 | 99.4 | 15 |
| LTR_DP19 | 1 | 98.3 | 34 |
| LTR_DP20 | 1 | 99.6 | 12 |
| LTR_DP21 | 1 | 99.4 | 10 |
| LTR_DP22 | 1 | 98.2 | 33 |
| LTR_DP23 | 2 | 96.0 | 7 |
| LTR_DP24 | 1 | 96.6 | 7 |
| LTR_DP25 | 1 | 99.0 | 10 |
| LTR_DP26 | 2 | 95.4 | 66 |
| LTR_DP27 | 1 | 99.7 | 19 |
| LTR_DP28 | 1 | 99.6 | 44 |
| LTR_DP29 | 1 | 99.8 | 7 |
| LTR_DP30 | 2 | 97.2 | 12 |
| LTR_DP31 | 1 | 99.1 | 46 |
| LTR_DP32 | 1 | 99.4 | 8 |
| LTR_DP33 | 1 | 98.6 | 24 |
| LTR_DP34 | 1 | 96.5 | 4 |
| LTR_DP35 | 1 | 97.4 | 2 |
| LTR_DP36 | 1 | 99.1 | 30 |
| LTR_DP37 | 1 | 99.4 | 3 |
| LTR_DP38 | 1 | 99.6 | 0 |
| LTR_DP39 | 5 | 89.0 | 82 |
| LTR_DP39 | 1 | 97.4 | 9 |
| LTR_DP41 | 1 | 91.2 | 19 |
