# Supplementary material for: De novo identification of LTR retrotransposons in eukaryotic genomes
Source: BMC Genomics. 2007 Apr 3;8:90. doi: 10.1186/1471-2164-8-90 (PMC1858694; doi:10.1186/1471-2164-8-90)
Supplement: Additional File 3 — Distribution of LTR retroelements in the C. elegans genome. The coordinates of elements are plotted with respect to their chromosomal locations. [file 1471-2164-8-90-S3.doc]

**
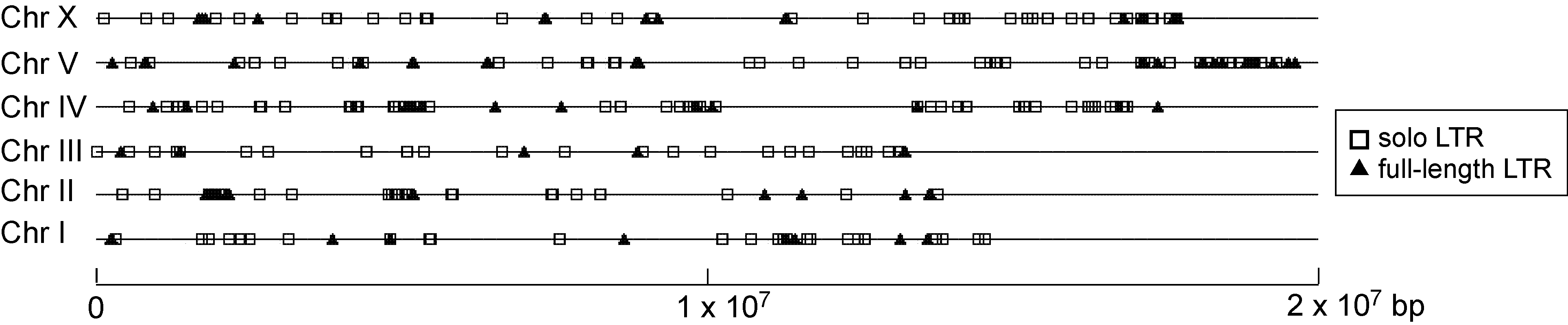
**

**Supplementary Figure 3:** Distribution of LTR retroelements in the *C. elegans* genome. The coordinates of elements are plotted with respect to their chromosomal locations.
